# Genotypes and subtypes of hepatitis C virus in Burundi: a particularity in Sub-saharan Africa

**DOI:** 10.11604/pamj.2014.19.69.4580

**Published:** 2014-09-24

**Authors:** Rénovat Ntagirabiri, Jean Dominique Poveda, Annie Mumana, Hermelance Ndayishimiye

**Affiliations:** 1 Centre des Maladies Digestives et du Foie « CEMADIF », Bujumbura, Burundi; 2 Laboratoire Cerba, Paris, France

**Keywords:** Hepatitis C virus, genotype, subtype, genotype 4

## Abstract

**Introduction:**

Hepatitis C virus (HCV) infection is a major public health issue. HCV genotype identification is clinically important to tailor the dosage and duration of treatment. Indeed, distinct therapeutic approaches are required for each genotype. Up to now, there is no study assessing HCV genotypes and subtypes in Burundi. The aim of the study was to determine HCV genotypes and subtypes in Burundi and to highlight the difficulties related to LiPA Method, widely used for African samples.

**Methods:**

In this study, a total of 179 samples contained anti-HCV antibodies were tested for HCV RNA, genotyping and subtyping. The analysis had been made in Cerba laboratory, Paris, France.

**Results:**

166 patients (92.7%) were genotype 4; 10 patients (5.6%) were genotype 1 and 3 patients (1.7%) were genotype 3. It was possible to determine subtypes for 51 HCV-4 (30.7%) patients. Among these, 25 (49.1%) had 4h subtype; 11 (21.6%) had 4e subtype; 2 (3.9%) had 4k subtype and 13 patients (25.5%) had 4a/4c/4d subtype. The LiPA method failed to subtype 115 (69.3%) HCV-4 and to separate the three subtype: 4a, 4c and 4d.

**Conclusion:**

Genotype 4 and subytype 4h followed by 4e are the widespread in Burundi.

## Introduction

Hepatitis C virus (HCV) infection is a major public health issue. Global prevalence of HCV is approximately 180 million people [1]. Central Africa has the second highest HCV prevalence: 6.0% of adults overall [2]. In Burundi, HCV prevalence is estimated up to 8.2% [3]. There are at least six genotypes throughout the world, each of them containing subtypes, according to the nucleotide divergence [4]. HCV genotype identification is clinically important to tailor the dosage and duration of treatment because distinct therapeutic approaches are required for each genotype [5]. Duration of treatment for a maximal sustained virological response (SVR) rate depends on genotype. In recent years, there has been an increasing interest in HCV subtype for SVR. Indeed, the subtype 1a could be associated with a lower response to anti-HCV therapy than subtypes 1b, 4a, and 4d [6, 7]. Up to now, there is no study assessing HCV genotypes and subtypes in Burundi. A further issue is that the laboratory techniques are validated based on American and European samples and could not be well adapted to african samples. The aim of our study was to determine HCV genotypes and subtypes in Burundi and to highlight the difficulties related to LiPA Method, widely used, about African samples.

## Methods

**Study population:** The study population consisted of consecutive willing patients aged ≥15years; attending medical cares in CEMADIF since January to May 2013. We included patients with anti-HCV antibodies positive. We excluded patients under interferon treatment or who have achieved the treatment with a SVR.

**Study design:** This was a cross sectional study. Socioeconomic and clinical data were collected by interview followed by a clinical examination done by a physician. After, patients were directed to the laboratory for blood sampling. In CEMADIF laboratory, all samples were collected and frozen by 4 hours. Molecular analysis was made by Cerba Laboratory, Paris, France. Samples were tested for HCV-RNA, HCV genotyping and subtyping. The quantification of HCV-RNA was made by the rt-PCR Taqman Roche method, with a sensitivity of 15 UI/ml (1.2log) and linearity of 15-100,000,000 UI/ml (1.2-8log) or by the Abbott RealTime HCV assay technique. The determination of genotype and subtype of HCV was made by rt-PCR technique and reverse-hybridization Line Probe Assay (LiPA) with the reagent SIEMENS VERSANT LiPA HCV2^®^ or by sequencing. Data were collected initially in a specialized data collection form, introduced into a Microsoft Excel worksheet, and finally transferred to the statistical package for social sciences (SPSS) version 10.0 for analysis.

**Ethical consideration:** Prior to blood sampling, the study was explained by a physician to the patient and a written informed consent was obtained. The tests were free of charge. The national ethics committee of Burundi approved the study.

## Results

During the period of the study, a total of 179 samples containing anti-HCV antibodies, from 179 consent patients, were collected and tested for HCV-RNA, HCV genotyping and subtyping.

**Figure 1:**
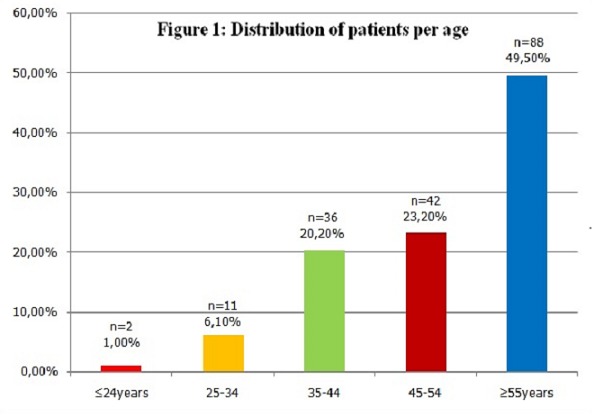
Distribution of patients per age

**Patient's characteristics:** A total of 105 (58.6%) females and 74 (41.4%) males had been enrolled. The mean age was 51.4 year (22 years to 73 years). The Figure 1 illustrates the distribution of patients per age. 123 (68.7%) were business men, 90 (50.3%) had a high school certificate and 55 (30.7%) had a bachelors degree. 122 (68.1%) were married while 31 (17.3%) were widows (ers). The patients were native of all provinces of the country. 103 (57.5%) resided in Bujumbura City. The HCV RNA was detected among all patients with the extremes of 113UI/ml (2.05log) and 20 208194 UI/ml (7.30log). Table 1 illustrates the viral load (VL) according to VHC-4 subtypes.

**Table 1:** Characteristics of patients according to subtype 4

Subtype	Mean age ± SD	VL < 800000UI/ml	VL > 800000 UI/ml	Medium VL (UI/ml)
4	50.89 ± 11,78	37.0% (n = 43)	63.0% (n = 72)	2 805 026
4e	53.22 ± 16,05	48.0% (n = 12)	52.0% (n = 13)	1 341 843
4h	51.20 ± 12,46	73.0% (n = 8)	27.0% (n = 3)	988 818
4k	63.00 ± 14,14	50.0% (n = 1)	50.0% (n = 1)	541 760
4a/4c/4d	52.86 ± 13,73	46.2% (n = 6)	53.8% (n = 7)	2605918
Global	51.56 ± 13,12	42.2% (n = 70)	57.8% (n = 96)	1418248

VL = viral load, SD = standard deviation

**Genotypes and subtypes:** 166 patients (92.7%) were genotype 4 (HCV-4), 10 patients (5.6%) were genotype 1, and 3 patients (1.7%) were genotype 3. Table 2 illustrates different genotypes and subtypes. Indeed, it was possible to determine subtypes for 51 HCV-4 (30.7%). Among these 51 patients, 25 (49.1%) had 4h subtype, 11 (21.6%) had 4e subtype, 2 (3.9%) had a subtype 4k and 13 patients (25.5%) had 4a/4c/4d subtype. The LiPA method didn′t allow to classify to one or other of these three 4a, 4c, 4d subtypes. For the 115 remaining HCV 4 (69.3%) it was not possible to determine subtype. Otherwise, some patients were erroneously classified by the LiPA technique in subtype 3k. For genotype 1, the subtype was specified only for one patient as 1a.

**Table 2:** Distribution of genotypes and subtypes in Burundi

Genotype (n)	Subtype	Number of patients	Percentage
Genotype 1 (n = 10)	1	9	88.9%
	1a	1	11.1%
Genotype 3 (n = 3)	3	2	66.7%
	3k	1	33.3%
Genotype 4 (n = 166)	4	115	69.3%
	4h	25	15.1%
	4e	11	6.6%
	4k	2	1.2%
	4a/4c/4d	13	7.8%

## Discussion

Our study indicates that HCV-4 is the most widespread HCV genotype in Burundi. This result can be extrapolated somehow to general population because patients were native of all provinces of the country. Our HCV-4 prevalence results in Burundi (92%) are also reported in Egypt (90%), Gabon (97%), Central Africa Republic (100%), Democratic Republic of Congo (100%), Liberia (100%), Uganda (100%), and Rwanda (100%) [8–13]. In other countries of central Africa, the rates of HCV-4 are heterogeneous. For example in Cameroon, the HCV-4 prevalence is 31% [11].

Another important finding is that the 4h and 4e subtypes are the most common in our country. The most common subtype in Egypt is 4a, in Gabon 4c and in Cameroun 4f [11, 12]. Table 3 shows different subtypes in different countries [11–19]. These findings further support the idea that the origin, evolution, and dynamics of HCV-4 are difficult to determine, given the limited population-based epidemiological studies and phylogenetic analysis of this genotype in the geographic regions in which it is endemic, such as Burundi, Egypt and Central or West Africa. However, the results of this study corroborate the information available from small-scale field surveys from Central African countries and Cameroon that HCV-4 strains circulating in Central Africa and Cameroon are extremely heterogeneous and that the estimated date of the most recent common ancestors for HCV-4 was 1500 (95% CI: 1350-1700), suggesting that it probably has been endemic for a long time [13–16, 20–25].

**Table 3:** Distribution of HCV-4 subtypes in different countries

Country	HCV-4 (%)	HCV-4 Subtypes (%)
Egypt	90	4a (55), 4 (24), 4o (7), 4m (3), 4l (3), 4n (2)
Gabon	97	4e (57,3), 4c (9,9), 4f (9,9) 4t(5,2), 4k(4,7), 4r (1,9), 4g (1,4)
Central African Republic	100	4 (66.7), 4k (33.3)
Democratic Republic of Congo	100	4 (30), 4c (30), 4k (24), 4r (14), 4a (5)
Cameroon	36	4f (22), 4 (5), 4t (5), 4k (5), 4e (1.4), 4o (1), 4p (1)
Liberia	100	4 (100)
Uganda	100	4 (66.7), 4r (33.3)
Tanzania	50	4 (100)
Rwanda	100	4k (100)
Sudan	5	4, 4e, 4c/4d
Tunisia	11	4k (5), 4a (3.6), 4 (2.6)
France	4–10	4d (2.3), 4a (2.2)
Italy	8.3	4d (5.9), 4 (2.4)
Spain	3–10	4c/4d (76.8), 4 (11.5), 4a (7.2), 4e (4.3)
Greece	13.2	4a (78)
Algerian	1.1	4a, 4a/4c/4d
Lybia	32,6	4
Saudi Arabia	60-80	4a (48.4), 4d (39) , 4n (6.25), 4m/4l/4r/4o (6.25)

According to the age of patients, our findings show an increasing prevalence with age. It seems possible that these results are due to mass vaccinations between 1940 and 1970 in Burundi and to ancestral practices such as scarification and circumcision. Some authors in Cameroon and Egypt reported the same factors. Indeed, an exponential spread of genotype 4 between 1920 and 1960 was detected in Cameroon, which coincided with the mass campaign against trypanosomiasis and mass vaccinations [13, 14, 23]. This great genetic diversity in sub-Saharan Africa might lead to the hypothesis that HCV-4 originated and propagated in Central and West Africa before spreading to other regions [13, 19–21].

It seems also important to emphasize that the LiPA method was unable to subtype a number of HCV-4 in our study. This finding was unexpected and suggests that the LiPA method, commonly used throughout the world, could be not adapted to African samples, particularly in countries where HCV-4 is predominant. Likewise, other authors have reported the same difficulty to subtype HCV-4, but in lower level than in our study [11]. The producers of reagents should be aware some more to guarantee a large diagnostic efficiency. At last, another interesting finding was that the genotype 3, not reported in the countries of eastern and central Africa, is however, found at 1.7% in our study.

## Conclusion

The genotype 4 is the most frequent in Burundi. The subtype 4h seems to be predominant. Further, the genotype 3 rare in central Africa, is found in our country at 1,7%. The producers of reagents should be aware some more to assure a large diagnostic efficiency.
